# Crucial timing in schizophrenia: role of DNA methylation in early neurodevelopment

**DOI:** 10.1186/s13059-014-0495-y

**Published:** 2014-10-29

**Authors:** Joanne Ryan, Richard Saffery

**Affiliations:** Cancer and Disease Epigenetics, Murdoch Children’s Research Institute, Royal Children’s Hospital, University of Melbourne, Parkville, VIC 3052 Australia; Department of Paediatrics, University of Melbourne, Parkville, VIC 3052 Australia; Inserm U1061, Hopital La Colombiere and Universite Montpellier 1, Montpellier F-34093, France

## Abstract

An exciting recent study examining the methylation profile of human brain tissue implicates early-life epigenetic disruption in the neurodevelopmental origin of schizophrenia.

See related research, http://genomebiology.com/2014/15/10/483

Schizophrenia is a debilitating neuropsychiatric disorder, with a lifetime prevalence of close to 1%, characterized by frequent psychotic episodes. There is a strong hereditary component that, until recently, remained largely unexplained, hampered by the involvement of a large number of gene variants with small effects, as well as rarer genes and copy-number variants with substantial effects. The largest and most recent genome-wide association study (GWAS) of 35,000 schizophrenia cases, and three times as many controls, has identified 83 novel loci contributing to overall risk, as well as 25 genes that had previously been implicated in the disease [1].

Although the peak onset of schizophrenia is in the adolescent and early-adult years, many believe this to be a disorder of neurodevelopment, with etiological origins much earlier in life. Indeed, it is thought that abnormalities occurring during the very early stages of development, caused by both environmental and genetic factors, trigger a cascade of processes cumulating in the emergence of symptoms at a later age. This neurodevelopmental hypothesis is supported by the findings of epidemiological studies linking early-life stress exposures with later risk of disease, as well as genetic association studies indicating the involvement of key genes in brain development. However, no study has yet provided insights into the possible molecular mechanism by which these effects could occur. Epigenetic variation is known to be susceptible to the environment, and insults occurring during neurodevelopment could conceivably leave a lasting impact on the epigenome and later phenotype.

In this issue of *Genome Biology*, Pidsley and colleagues [2] identify distinct methylomic profiles in the prefrontal cortex (PFC) of the adult schizophrenic brain, which are replicated in an independent sample. Furthermore, by comparison with early-life human fetal brain samples, the authors demonstrate that several schizophrenia-associated differentially methylated probes map to genes with roles in fetal brain development. These findings thus provide compelling support for the neurodevelopmental hypothesis of schizophrenia and suggest, for the first time, a role for early-life epigenetic disruption in mediating these effects [2].

## Epigenetic analysis: peripheral biomarkers versus central nervous system disease-associated variation

Given the likely involvement of both genetic and environmental components to disease risk, increasing research has focused on the role of epigenetics in the pathophysiology of schizophrenia. As with many complex disorders, recent work has taken a ‘hypothesis-free’ epigenome-wide approach to the study of DNA methylation. Broadly speaking, these studies fall into two categories: those investigating methylation in peripheral tissue of living individuals and those examining DNA methylation in post mortem central nervous system (CNS) tissue.

A comparison of leukocyte DNA methylation between 24 medication-naive schizophrenia cases and 23 controls using the Illumina Infinium HumanMethylation450 array platform identified 234 disease-associated genomic sites of differential methylation [3]. These were subsequently replicated in a cohort of three monozygotic twin pairs discordant for the disorder. A more recent study combined affinity purification of methylated DNA and deep sequencing of blood from 759 schizophrenia cases and 738 controls [4]. A number of significant sites of differential methylation were identified, including *FAM63B*, implicated in neuronal differentiation, and *RELN* (encoding the serine protease reelin), the most widely studied candidate gene in methylation studies. Importantly, replication of key findings was carried out in an independent case–control sample.

Although blood-based studies can reveal biomarkers of schizophrenia, their relevance to disease etiology is questionable, given the tissue-specific nature of epigenetic profiles. Thus, studies of post mortem CNS tissue, such as that undertaken by Pidsley and colleagues [2], are very important from an etiological perspective. A number have been previously reported, although replication of individual findings is unfortunately limited, most likely owing to small sample sizes, different study populations and inconsistent methodological approaches.

The first foray into the area was reported in 2008, using post mortem brain tissue of 35 individuals with schizophrenia and 28 matched controls [5]. Data were generated for 12,192 CpG islands by hybridization to the unmethylated fraction of genomic DNA. Considerable evidence for epigenetic changes in association with schizophrenia was reported, with a number of loci involved in neuronal development, in addition to glutamatergic and GABAergic genes.

More recent studies have carried out epigenome-wide association studies (EWAS) using the robust Illumina Infinium HumanMethylation BeadChip platform. The first-generation 27K arrays were used to profile DNA methylation in 216 PFC samples, including 97 schizophrenia cases, in conjunction with genome-wide single-nucleotide polymorphism (SNP) analysis. A total of 107 disease-associated sites of differential methylation were found, the majority of which showed increasing methylation in disease. Interestingly, a number of *cis*-methylation quantitative trait loci (QTLs - SNPs associated with DNA methylation levels at specific genomic sites) were also identified, including at SNPs previously associated with schizophrenia [6]. For example, the A allele of rs6311, previously linked to schizophrenia, was associated with higher methylation at the serotonin receptor (*HTR2A*) gene encoding 5-hydroxytryptamine receptor 2A.

The next-generation Infinium HumanMethylation450 BeadChip platform has recently been used by Wockner and colleagues [7] to profile DNA methylation at over 485,000 sites in post mortem frontal cortex tissue from 24 schizophrenia patients and 24 controls. A total of 4,641 schizophrenia-associated differentially methylated probes were identified after adjusting for age and post mortem interval. Nearly half were hypomethylated in the schizophrenia group, representing 2,929 unique genes [7]. Interestingly, approximately 17% of these matched a previous list of differentially methylated genes reported in peripheral blood of schizophrenia subjects [8].

## The Pidsley study

Cross-sectional EWAS studies, although highly informative, provide only limited information regarding schizophrenia etiology and cannot rule out reverse causation as a source of the observed differential methylation. Indeed, it is likely that many reported methylation changes in the brain of schizophrenia subjects result from the disease process itself, or associated factors such as medication use. This is where the current work of Pidsley and colleagues stands alone. In addition to performing an EWAS for schizophrenia *per se*, they have explored the ontogeny of methylation loci of interest in the early fetal brain in order to gauge the likelihood of disease-associated methylation variation that has origins in early life.

Using the Infinium HumanMethylation450 BeadChip platform, they first compared more than 485,000 CpG sites across the genome in brain tissue from the PFC and cerebellum of 20 schizophrenia patients and 23 age- and gender-matched controls. Importantly, cell heterogeneity was considered during the analysis. A number of PFC-specific sites of differential methylation were found between patients and controls, with four gene-associated probes reaching genome-wide significance levels. The gene *GSDMD* (encoding gasdermin-D) showed, on average, 4.4% increased methylation in patients, whereas the GTPase-activating protein gene *RASA3*, *PPFIA1* (encoding liprin-alpha-1) and *HTR5A* had decreased methylation in patients compared with controls (effect sizes ranging from a mean of 2.3% to 8.7%).

Using an alternative approach of data aggregation across adjacent probes, regions of coordinated differential methylation were also identified, including an 8-kb block of 28 hypomethylated probes (average decrease of 1.8% between cases and controls) within the *NRN1* gene body (encoding neuritin). Genetic variation in *NRN1* has previously been associated with schizophrenia [9], and it is known to play a crucial role in neurodevelopment and synaptic plasticity. Importantly, all of the significant differences were specific to the PFC, a brain region known to be functionally important in the etiology of schizophrenia.

The authors validated their findings by using bisulfite sequencing of three differentially methylated regions near genes *NRN1*, *C8A* and *RASA3* and also performed a replication analysis by using PFC samples from a second brain-bank sample consisting of 18 schizophrenic patients and 15 controls. They found surprisingly strong correlations between differentially methylated sites in the two cohorts (*r* =0.54) and, importantly, were able to replicate the significant hits from their initial discovery set. Weighted gene co-methylation network analysis was employed, and 12 modules in the PFC were significantly associated with schizophrenia. Of those, two showed particularly strong negative associations with the disease, which were conserved across the two cohorts, and again were absent from the cerebellum. Consistent findings from both pathway and gene ontology analysis indicated that the modules were enriched for functions involved in the development and function of the nervous system.

Breaking new ground, Pidsley *et al*. then sought to determine whether the differentially methylated CpG sites associated with schizophrenia were susceptible to dynamic methylation changes during brain development. To perform this analysis, they used a sample of 179 human fetal cortex samples, aged between 23 days and 6 months post-conception. Remarkably, the authors found that 44% of the previously identified schizophrenia-associated sites of differential methylation were dynamic in the brain across pregnancy, thus providing strong new evidence in support of a neurodevelopmental origin for schizophrenia that is associated with early-life epigenetic disruption in the developing brain. For example, one of the top-ranked schizophrenia-associated differentially methylated probes, in the gene body of *MYT1L*, is significantly hypomethylated in cases relative to controls and is also highly dynamic in association with neocortical development. *MYT1L* encodes a potent myelin transcription-factor-like protein integral to neurodevelopment.

## Replication, replication, replication

A limitation of the Pidsley study is that only limited comparisons with previous data were made. A goal for the future should be the use of a single methylation platform by multiple teams to examine post mortem brain biopsies, which will facilitate a direct comparison of findings across different studies. Nonetheless, several of the genes showing differential methylation in this study have also recently been linked to schizophrenia as part of a large international GWAS initiative [1], including calcium channel subunit *CACNA1C*, the t-SNARE domain-containing protein *TSNARE1*, the phosphatidylinositol transfer protein *PITPNM2* and the leukocyte antigen *HLA-L*. Perhaps just as important as the reported findings is what can be found buried within supplementary data that highlights common ‘hits’ in at least two, if not more, instances. For example, the kinesin light chain-1 gene *KLC1* shows altered DNA methylation in Pidsley *et al*. [2] and Wockner *et al*. [7] and is also located within one of the 108 genomic loci associated with schizophrenia by GWAS. The *MYT1L* gene is also differentially methylated in both epigenetic studies. Furthermore, whereas direct replication across studies is not apparent for many genes, consistent classes of gene families are reported across multiple genetic and epigenetic analyses. This includes multiple calcium channel and serotonin transporter genes. Numerous other overlaps exist, although it is important to point out that the majority of ‘hits’ in any one study are not present in any other.

## Concluding remarks

This study presents a novel approach to tackling one of the most problematic areas of epigenetic analysis of human disease, namely how to determine the role of early-life epigenetic disruption in neuropsychiatric illness. The evidence reported by Pidsley and colleagues supports a role for epigenetic variation during neurodevelopment as a contributor to schizophrenia - but remains indirect - despite being robust across two independent cohorts. Nevertheless, data continue to mount from other studies in support of the underlying hypothesis, with emerging evidence implicating disruption of multiple cellular pathways in disease etiology. Technology in this field is rapidly advancing, and there are many international repositories of post mortem brain material yet to be examined. As such, we can expect to see many more studies using higher-density methylation profiling in the near future. However, the now-conclusive role of underlying genetic variation in determining the methylation status of many genomic loci necessitates future studies combining both genetic and DNA methylation data into a single analysis approach. Finally, although exploring DNA methylation is a logical first step, such studies should also be expanded to include other epigenetic mechanisms, including non-coding RNAs and chromatin modifications.

In summary, mounting epigenetic data appear to be converging on common pathways and processes in the etiology of schizophrenia, at least some of which are likely to be disrupted during early development. Although individual studies are beginning to yield compelling findings, the data are as yet inconclusive. International coordination of tissue sample availability and EWAS analysis, in a manner similar to that which has proven so successful for GWAS studies, represents the most promising approach towards understanding the role of epigenetic disruption in schizophrenia and other neuropsychiatric disorders. Such efforts are already beginning to yield exciting results in Alzheimer’s disease [10].

## Abbreviations

EWAS: Epigenome-wide association study
GWAS: Genome-wide association study
PFC: Prefrontal cortex
QTL: Quantitative trait locus
SNP: Single-nucleotide polymorphism
